# t-RNA mediates provirus deletion in HIV-infected cells

**DOI:** 10.1186/s12977-025-00667-0

**Published:** 2025-07-01

**Authors:** Corrado Gurgo, Genoveffa Franchini

**Affiliations:** Animal Models and Retroviral Vaccines Section, National Cancer Institute, National Institutes of Health, Bethesda, MD 20892 USA

**Keywords:** Linear tRNA, Latency, Deletion, Integration

## Abstract

**Background:**

In the early phase of HIV infection, as studied in vitro, high levels of unintegrated (both linear and circular) and integrated (provirus) forms of viral DNA are seen, and cells produce high levels of virus. In time, the level of unintegrated DNA declines, followed by a progressive decline in virus expression. Extensive studies of the proviral landscape in people living with HIV (PLWH) on antiretroviral therapy (ART) show that only about 2% of proviruses are intact; the remainder are characterized as defective and contain numerous deletions of proviral DNA segments and hypermutations. In the current study, we investigated the decline of viral expression in infected T cells in search of mechanisms involved in proviral inactivation.

**Results:**

We derived clonal lines from Jurkat cells infected with HIV MN and monitored them for viral expression over time in culture. In a subset of clones that displayed a decline in expression, we found provirus containing large deletions and the integration of a retrotranscribed molecule of tRNA^Gly^ adjacent to the 3’-end of the proviral DNA. We provide evidence linking the proviral deletions to the insertion of a reverse transcribed tRNA^Gly^ molecule and propose a mechanism for its self-primed reverse transcription.

**Conclusions:**

Large deletions of proviral DNA have been reported in PLWH on ART and attributed to errors that occurred in the synthesis of the minus strand during the reverse transcription of the viral genome. Our results support an additional mechanism for proviral deletions, mediated by tRNA^Gly^, in the inactivation of the provirus.

**Supplementary Information:**

The online version contains supplementary material available at 10.1186/s12977-025-00667-0.

## Background

In vitro studies of HIV infection initially characterized the course of HIV infection as having two phases: acute and chronic. In the former, high levels of unintegrated (linear and circular) and integrated (provirus) forms of viral DNA are seen, and cells produce high levels of virus [1]. In time, the level of unintegrated DNA declines, followed by a progressive loss of viral expression as indicated by p24 release, signaling entry into the chronic phase. Analysis of latently infected clones by chromatin immune precipitation (ChIP) assay reported by Pearson et al. [2] suggested that the progressive shutdown of HIV transcription in vitro was caused by epigenetic modification of chromatin structure that selectively targets HIV transcription initiation.

The characterization of molecular events that occur during in vivo replication has been facilitated by the wide usage of potent inhibitors of HIV replication and powerful sequencing technologies. Early studies utilized an ex vivo viral outgrowth assay [3] to measure the ability of latently infected cells from people living with HIV (PLWH) undergoing antiretroviral therapy (ART) to produce virus upon stimulation [4]. Of 213 proviral clones unable to replicate and produce p24 in culture, about 88% were found to be defective upon analysis by PCR. The remaining 12% had intact genomes and normal long terminal repeat functionality but were not inducible by a single round of stimulation in vitro. Subsequent studies relying on sequencing of proviral DNA directly from PLWH who began ART during the chronic or acute phase of infection yielded an estimated 98% defective proviruses and 2% intact [5, 6]. The most common defects were deletions and hypermutations.

In regard to the mechanism(s) by which virus was rendered replication incompetent, Ho et al*.* [4] reported the following: deletions, potentially caused by copy choice recombination during reverse transcription as described by Sanchez et al. [7]; APOBEC3G-induced hypermutation [8]; and frameshift or nonsense mutations [9]. The analysis by Ho and colleagues excluded that the non-induced proviruses were silenced through CpG methylation at the 5’ LTR, a mechanism reported in some models of HIV latency [10] or integration into chromosomal regions repressive for transcription, thus implying that other factors must have prevented expression. A later publication reported that 97% of defective proviruses had defects affecting the transcriptional activator Tat [6].

While unable to generate infectious virus, defective provirus could still contribute to the pathogenicity of HIV. This was suggested by the work of Imamichi et al. [11, 12], who reported persistent defective provirus in PLWH capable of producing unspliced HIV RNA encoding chimeric proteins. The authors suggested that the protein products of these novel RNAs could be responsible for the persistent seropositivity to HIV in PLWH on long term ART by continuously stimulating the host immune system against “foreign” RNA and proteins.

Studies investigating various aspects of HIV infection routinely employ T cell lines. In our study using single cell clones derived from Jurkat cells infected with the HIV MN isolate [13], we found a provirus that was inactivated by large deletions, including a complete LTR, following the insertion of a reverse transcribed tRNA^Gly^ at the site of integration.

In addition to their role in decoding mRNA sequences, tRNA molecules are involved in other pathways and cellular processes [14, 15]. In our study reported here, we provide evidence that tRNA serves an additional role in mediating the deletion of provirus. Relevant to our finding is earlier work developed in the Berkhout laboratory [16]. The authors reported non-homologous recombination in HIV, mediated by a tRNA molecule adjacent to a hairpin structure of 300 nucleotides experimentally inserted in the nef region. In two replication competent variants, sequence analysis revealed the presence of 63 nucleotides (nt) of tRNA^Asp^ or 65 nt from tRNA^Glu^ in place of the deleted hairpin. The two tRNA sequences were subsequently removed due to a recombinant/deletion event.

Also of relevance is a study by Colicelli and Goff [17] describing the insertion of a retrotranscribed tRNA^Gly^ molecule between the LTRs of a cloned circular episomal form of MMLV. The authors provided a model for the insertion of tRNA^Gly^ based on the usage of this molecule as a reverse transcribed spurious primer that was linked to the viral RNA. In our model, the reverse transcription of tRNA^Gly^ is self-primed and independent from the reverse transcription of the viral RNA, and the insertion adjacent to the 3’-end of the provirus is not directly linked to the HIV integration.

## Methods

### Infection

The Jurkat T cell line and the MN strain of HIV-1 were utilized in this investigation. 2 × 10^6^ Jurkat cells (J20) were incubated at 37 °C for 1 h in 2 ml of cell-free culture supernatant of H9 cells infected with an early passage of HIV MN virus and gently shaken every 15 min. Thereafter, the supernatant was removed, and the cells were washed in phosphate buffered saline (PBS) and resuspended in 10% RPMI at a concentration of 1 × 10^5^ cells/ml. Medium was changed every 4–5 days and the level of supernatant p24 determined.

### ELISA

Cell free supernatants were analyzed for p24 using the Retro-Tek HIV-1 p24 Antigen ELISA kit by ZeptoMetrix (Buffalo, NY).

### Flow cytometry

Cells were washed 2 times in PBS (without Ca^++^ or Mg^++^) and stained with phycoerythrin (PE)-labeled monoclonal antibody against CD4 (clone OKT4; BD Biosciences, San Diego, CA; Catalog #566680). An irrelevant PE-labeled antibody was used as a control (clone L27; BD Biosciences; Catalog #346595). After 30 min at 4 °C, cells were washed two times in PBS and resuspended in 500 µl PBS. Fluorescence analysis was performed with a FACS scan flow cytometer (Becton Dickinson, San Jose, CA) equipped with a 488 nm argon laser. Cells were gated as FL2-PE-CD4 (OKT4) vs FL1-FITC-unstained (Fig. S2C).

### Luciferase infectivity assay

Samples of 2 × 10^5^ TZM-bl cells (a CXCR4-positive HeLa cell line expressing CD4 and CCR5 and containing the luciferase reporter gene under the control of the HIV LTR) were seeded in duplicate in a six well plate in 2 ml of DMEM containing 10% FCS and 1% pen/strep. Upon cell adhesion, medium was removed, and the cells were incubated for 5 h with 1 ml of a culture supernatant from the J20/MN clones. Thereafter, the supernatant was removed, cells washed three times with PBS and incubated at 37 °C in 2 ml of fresh medium. At the indicated times, cells were lysed and the luciferase activity determined using 10 µl of lysate and the kit and protocol of the Promega Luciferase Assay System. The TZM-bl cells were obtained from the NIH Research and Reference Reagent Program.

### DNA extraction

Total cellular DNA or genomic DNA were respectively prepared using the Qiagen (Germantown, MD) DNeasy Blood &Tissue Kit and the Puregene Core Kit A.

### Southern blot

Total cellular DNA was digested with EcoRI. The restricted fragments were separated by agarose gel electrophoresis, transferred onto Hybond N^+^ membranes, and hybridized with p32-labeled probes following standard procedures. A Hind-EcoRI and an EcoRI-BamHI fragment, respectively specific for the gag and env regions of HIV MN (GenBank: M17449.1) [13], were used for the detection of proviral or episomal DNA.

### Polymerase chain reaction (PCR)

The following PCR primers and probes were used for detection:

*Total DNA*. Forward primer: 5′-TGTGTGCCCGTCTGTTATGT-3′ (556–575); reverse primer 5′-GAGTCCTGCGTCGAGAGAGC-3′ (695–676); probe: 5′- CAGTGGCGCC-CGAACAGGGA-3′ (632–651). Cyclic parameters: 2’/94° (1 cycle initial denaturation); {30″/94° (denaturation); 30″/60° (annealing); 30″/72° (extension)} 35 cycles;10’/72° (final extension). The PCR products were analyzed by Southern blot.

*2-LTR.* Forward primer (3’-R/MN): 5′-AACTAGGGAACCCACTGCTTAAG-3′ (9604 → 9626); reverse primer (5’-U3/MN): 5′-CCCACAGATCAAGGATGTCTTGTC-3′ (51 → 28), and probe (3’-U5/MN): 5′- ACACTACTTGAAGCACTCAAGGCAAGCT-TT-3′ (9663–9634). The primers were used as previously described [18], adapted to the MN sequence. Cyclic parameters: 2’/94° (1 cycle initial denaturation); {30″/94° (denaturation); 30″/60° (annealing); 30″/72° (extension)} 35 cycles; 10’/72° (final extension). The PCR products were analyzed by Southern blot.

*Integrase gene.* Forward primer (MN-IN/5’): 5′-GGCATGGGTACCAGCACACAAAGG-3′ (4163 → 4186); reverse primer (MN-IN/3’): 5′-GCCAGTCTCTTTCTGGTGTATGC-3′ (5293 → 5271). Cycling conditions: 2’/94°; {30″/94°; 30″/55°; 1′30″/68°} 35 cycles; 10’/68°.

### Provirus integration

*Primers used for detecting proviral integration in clone A-1–26 by Alu PCR.* 5’-U3/MN primer (MH-536/MN): 5′-CCCACAGATCAAGGATGTCTTGTC-3′ (51 → 28) coupled with: 5′-ACTGCACTCCAGCCCTGGGCGAC-3′ (Alu forward). 3’-U5/MN primer: 5′-AACTAGGGAACCCACTGCTTAAG-3′ (9604 → 9626) coupled with 5'-TGCTGGGATTACAGGCGTGAG-3′ (Alu reverse). Cycling conditions: 2’/94°; {15″/94°; 30″/55°; 4’ /68°} 40 cycles; 10’/68°.

*Primers used for the amplification and sequence of the integration site of the 5’-LTR and the 3’-LTR in subclones of A-1–26.* 5’-U3/MN primer (as above) coupled with F8-R: 5′-CATAGAGGCCATTCCTTGGA-3′ (13548 → 13529 of clone GHc-528F8 from chromosome X). 3’-U5/MN primer (as above) coupled with F8-L: 5′-GAAAGAAGTGGGTTGGGACAA-3′ (13134 → 13154 of clone GHc-528F8). Cycling conditions: 2’/94°; {15″/94°; 30″/55°; 1’ /68°} 40 cycles; 10’/68°.

*Primer used for the detection and sequence of 5’ half provirus and 3’ half provirus:* Forward primer 5’-MN → F8-R: 5′- CAAACTTGGCAATGAAAGCA-3′ (5954 → 5936); reverse primer F8-R (as above); forward primer F8-left (as above), reverse primers (3’-MN → F8-L): GATACTTGGGCAGGAGTGGA (5727 → 5747).

## Results

In our approach, we isolated clones from an infected T-cell line expressing declining levels of p24 in chronic infection and compared their efficiency in sustaining p24 expression, CD4 positivity, episomal DNA, and provirus over time. We utilized a Jurkat T-cell line enriched in cells expressing CD4 (J20, > 99% CD4^+^) infected with an early passage of the uncloned MN strain of HIV (J20/MN). After 6 weeks of continuous growth, 49 clones were isolated, monitored for the expression of CD4 and p24, and frozen after one month (T1), three months (T2), and six months (T3) of culture (Fig. S1). Of these 49 clones, 20 were nonproducers. The remaining clones were categorized as high, moderate, or low producers based on the amount and persistence of viral antigen released (Fig. S2A, Tables S1 and S2). Despite the differences in productivity, all clones showed declining expression with time in culture. A 50% average decline in soluble p24 with respect to the peak value was shown with the high producer clones at the end of the observation period. The percentage of CD4^+^ cells was generally low in all clones (Fig. S2B and Table S3) but remained high (87%) in the uninfected J20 line after one year of continuous growth in culture.

Viral DNA was not detectable by Southern blot in the nonproducer clones, but it was detected by PCR in 12 analyzed clones, of which 7 were also positive for 2-LTR circles (Fig. S3). There was no evidence of integration in two randomly selected clones (A-1-20 and A-1-40) analyzed by Alu-PCR (A-1-20 results are shown in Fig. S4A). The latently infected ACH2 cell line was used as a positive control (Fig. S4B and S4C). As in all nonproducers, CD4 was strongly downregulated in these two clones, limiting the possibility that the 2-LTR circles resulted from infection. Production of 2-LTR circles in the absence of functional provirus can be explained by amplification of the episomal form as reported for 1-LTR circles [19, 20]. The failure to detect viral integration or the presence of 2-LTR circles with the 5’ and 3’ LTR specific primers used, as reported in this study, can be attributed to deletions in the LTR.

**Fig. 1:**
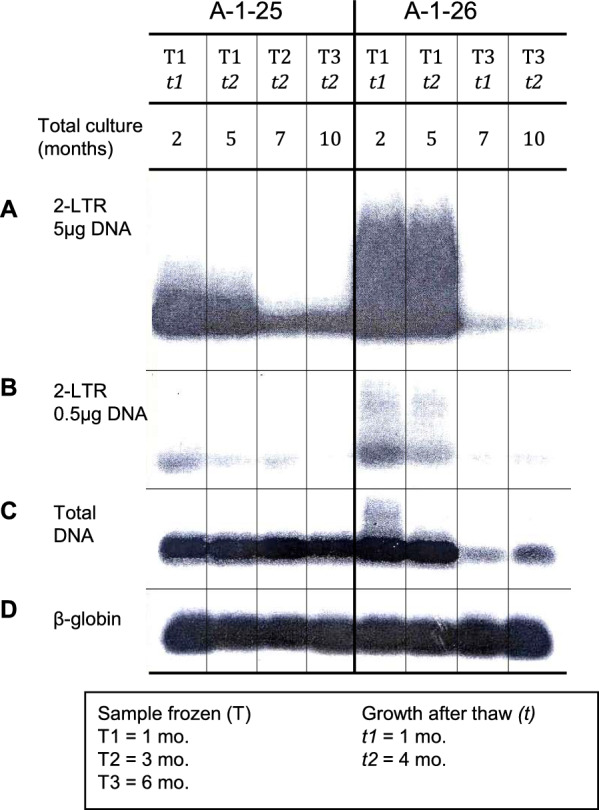
Progressive loss of HIV episomal forms in the transition from producer to non-producer state. Two clones were frozen at three timepoints: 1 month (T1), 3 months (T2), and 6 months (T3) in culture. Samples were thawed and analyzed at 1 month (*t1*) and 4 months (*t2*) of continuous growth for the presence of (**A**,**B**) 2-LTR, (**C**) total DNA, and (**D**) β-globin. The total time of culture (T + *t*) is indicated in the figure. The decline of total DNA over time reflects the disappearance of episomal DNA

**Fig. 2:**
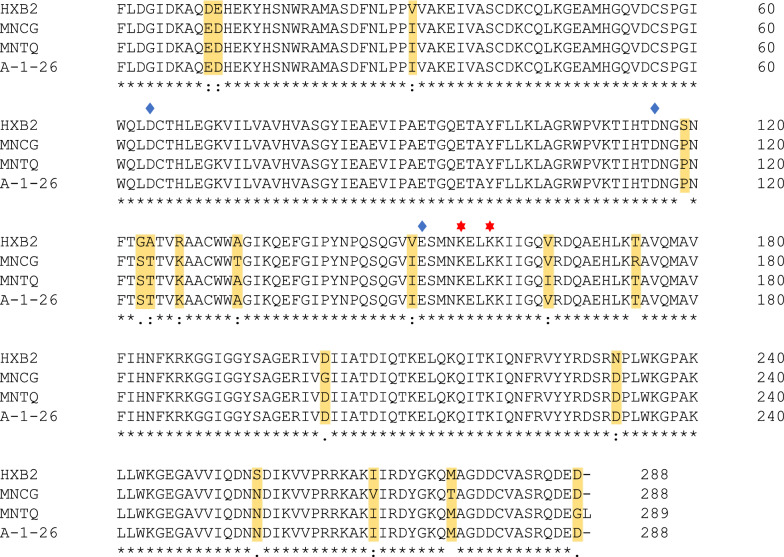
Sequence alignment of the integrase gene of A-1-26 and three HIV isolates. Clustal W sequence alignment of A-1-26 integrase with the integrase of HXB2 (GenBank Accession no: K03455) and of two HIV-1 isolates MNCG (M17449) and MNTQ (AF075719). Blue diamonds and red stars indicate the highly conserved amino residues (D64/D126/E152) and (K56/K59) respectively marking the catalytic site and the DNA binding site of the IN. Sequence differences between isolates are highlighted. Asterisk (*) indicates identical residues. Conservative and semiconservative substitutions are respectively indicated with a colon (:) and period (.)

Having observed that all producer clones show a decline in p24 expression with time in culture, we focused on two clones, A-1-25 and A-1-26, from the group of moderate producer clones representing an intermediate stage between high and low producers. The clones were thawed to generate the respective T1, T2, and T3 lines (Fig. S1). PCR analysis consistently showed the presence of 2-LTR circular forms during the release of p24 into the culture supernatant: the decline of p24 paralleled that of the circular forms (Fig. 1, Tables S4 and S5). Because proviral DNA was not detectable in these clones by Southern blot or Alu PCR in long-term cultures, we sequenced and confirmed the presence of a functional integrase (IN) in an early culture of A-1-26 (Fig. 2). Sequence alignment of the IN gene of this clone with the sequences of three published molecular clones of HIV showed a limited number of variations. The catalytic triad (D64, D116, and E152) and K156 and K159, essential for binding and integration of proviral DNA, were highly conserved (Fig. 2).

**Fig. 3:**
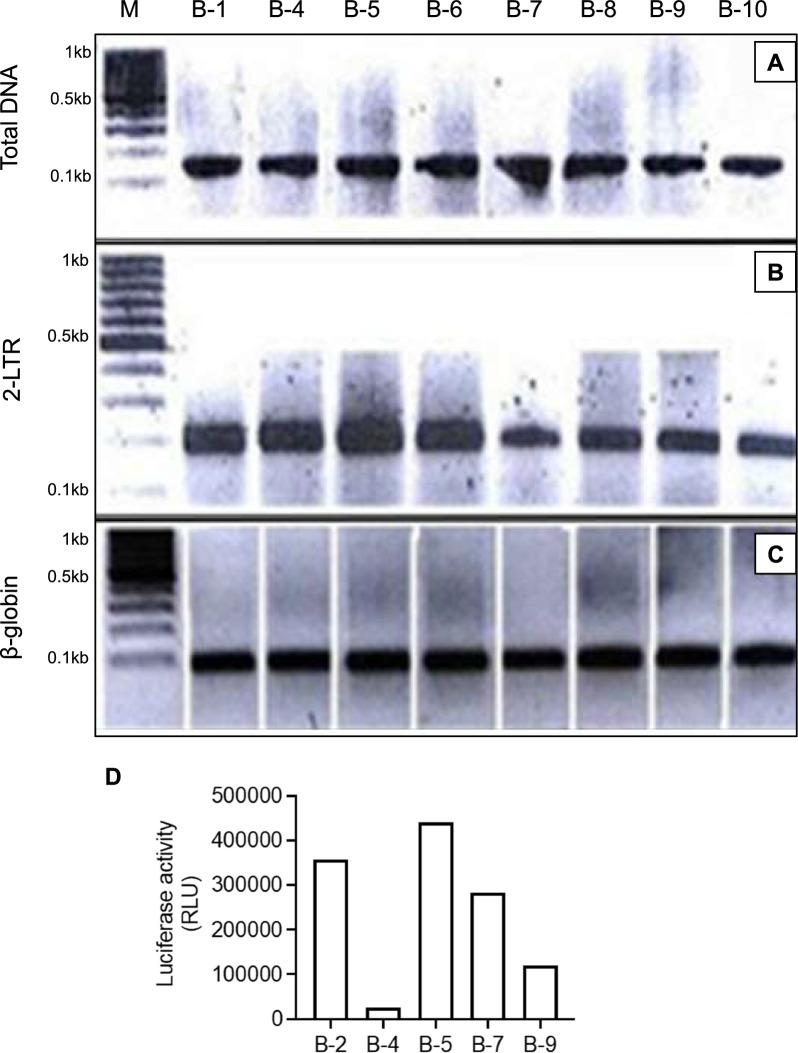
Presence of viral DNA in A-1-26 subclones. **A**–**C** Presence of viral DNA was determined by Southern blot analysis of the PCR amplification of (**A**): a highly conserved region in the gag gene (nt 556 → 695); (**B**): 2-LTR circles; and (**C**): the β-Globin gene used as the housekeeping gene, with primers and probe reported in Material and methods. The size of the bands is as expected from the primer utilized. **D** Luciferase activity induced by the supernatant of several subclones of A-1-26 after one month of growth from a single cell

To rule out the possibility that the decline of p24 expression and viral DNA observed in the two moderate producer clones A-1-25 and A-1-26 was due to the outgrowth of uninfected cells, eight subclones of A-1-26 (B-1, B-4 to B-10) were analyzed for the presence of 2-LTR circular forms, total viral DNA, and the ability of their supernatants to induce luciferase activity in the TZM indicator line. The results showed that all subclones were positive for viral DNA (Fig. 3A–C), but only a few clones expressed some level of infectious virus at one month of growth from a single cell (Fig. 3D).

**Fig. 4:**
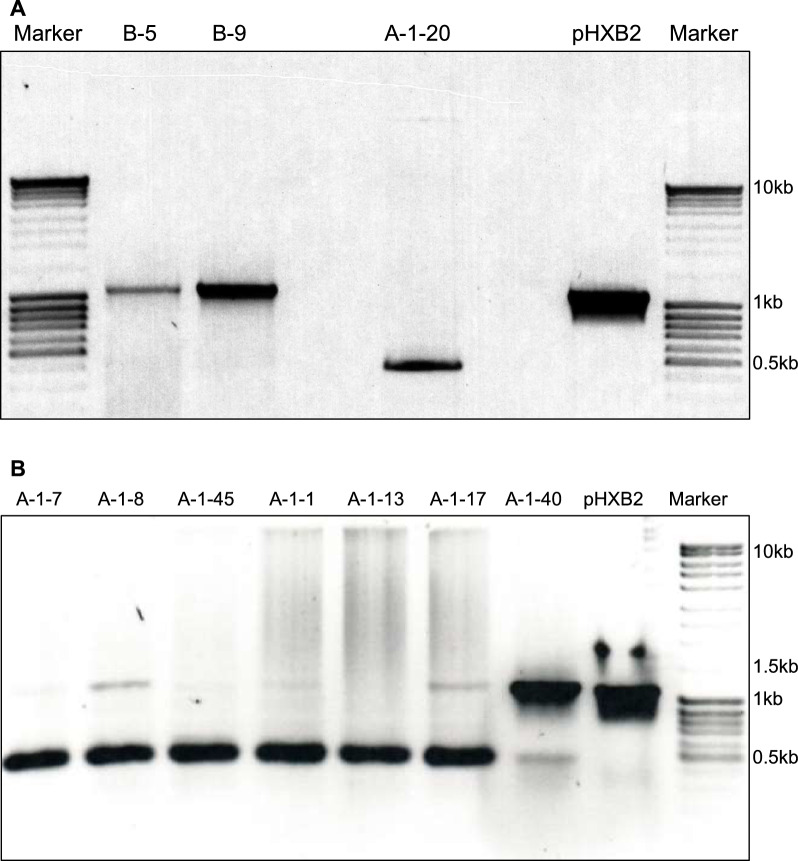
PCR amplification of the IN gene in clones of J20/MN. A fragment of 1.1 kb consistent with the presence of the intact IN gene was amplified in two subclones of the moderate producer clone A-1-26. A ~ 0.5 kb fragment was detected in the nonproducer, provirus negative clone A-1-20. **B** Analysis of the IN gene extended to seven nonproducers shows a thick ~ 0.5 kb band in six clones, three of which additionally showed a faint 1.1 kb band. A thick band of the expected 1.1 kb size and faint band of ~ 0.5 kb were found in one clone (A-1-40)

Subsequently, we analyzed two subclones (B-5 and B-9) of the moderate producer A-1-26 and the nonproducer clone A-1–20 for the presence of the IN gene. B-5 expresses infectious virus as confirmed by the luciferase infectivity assay (Fig. 3D) and transmission to Jurkat cells (Fig. S5); B-9 was negative by luciferase assay. A 1.1 kb fragment was observed in the DNA of both A-1–26 subclones, as expected for the amplification of the IN gene. A ~ 0.5 kb fragment was observed for the IN of clone A-1–20 (Fig. 4A). We extended this analysis to other nonproducer clones. PCR analysis in seven nonproducer clones of J20/MN showed a thick 1.1 kb band and a faint ~ 0.5 kb band in one clone (A-1–40). A thick ~ 0.5 kb band was observed in the other six clones, two of which additionally showed a faint 1.1 kb band. Agarose gel electrophoresis of the amplicons of the IN gene of seven randomly selected clones (from twenty nonproducers) highlighted a progressive deletion in the viral gene, suggesting the involvement of a cellular mechanism in the progressive shutdown of virus expression (Fig. 4A, B).

## tRNA^Gly^/provirus interaction

**Fig. 5:**
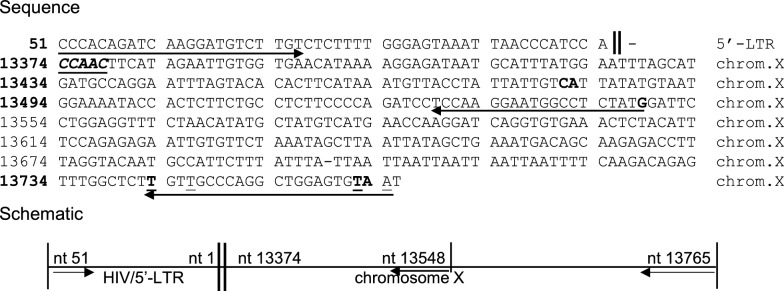
Detection of integration of the 5′-LTR of HIV MN in the B5 subclone of A-1-26 by Alu PCR. The amplified fragment contains the first 51 nt of the 5′-LTR and 391 nt of chromosome X. Sequence differences between the Alu primer and the corresponding amplified sequence are underlined. PCR was repeated using a primer (F8-R) closer to the detected integration site. Forward primer: 5′-U3, nt 51 → 28. Reverse primers: F8-R, nt 13548 → 13529; 3′-Alu (nt 240 → 261, consensus sequence) nt 13765 → 13743

*5’- LTR integration site.* We analyzed subclone B-5 for the presence of provirus by Alu PCR. A PCR fragment of 0.45 kb, obtained with the forward primer 5’-U3/MN and the reverse 3’-Alu primer, was TA cloned and sequenced. BLAST analysis of the sequence of the amplified fragment showed that the provirus was integrated on chromosome X in an intron of the dystrophin-related protein 2 gene, located between nt 13373 and 13374 of the human DNA sequence Z68331.3 (Fig. 5), in reverse orientation with respect to the direction of gene transcription.

**Fig. 6:**
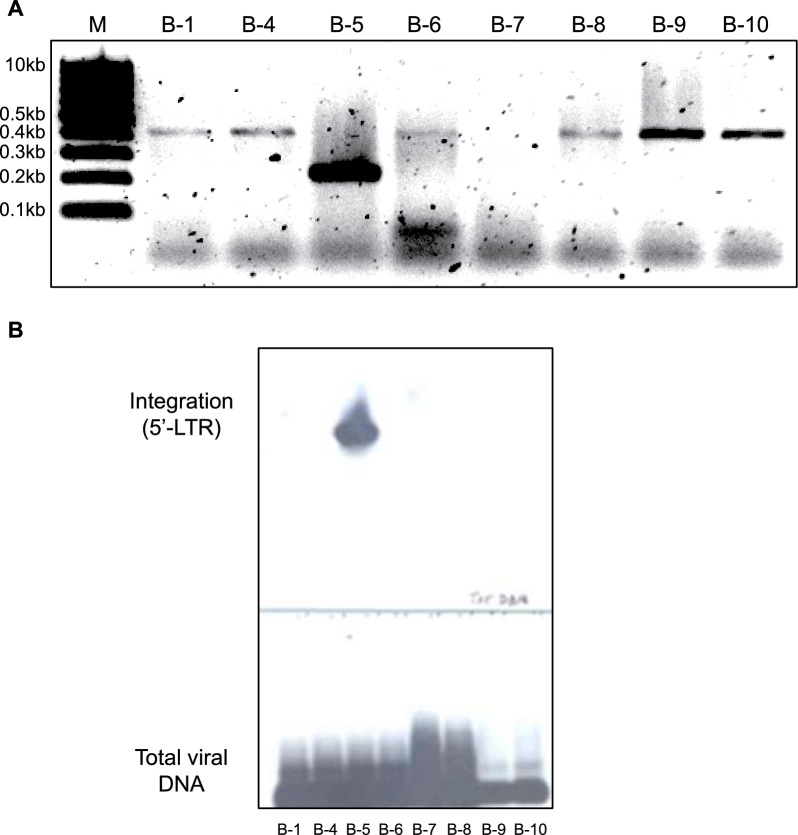
Analysis of the integration site of HIV MN in subclones of A-1-26. **A** Agarose gel analysis of DNA fragments amplified by Alu PCR for the detection of provirus in eight A-1-26 subclones shows the expected fragment of ~ 0.26 kb in subclone B-5, and a 0.4 kb fragment in 7 of the remaining subclones. **B** Detection of integration in one A-1-26 subclone. Integration in subclone B-5 is confirmed by Southern blot. All examined subclones were positive for the presence of total viral DNA. Sequence analysis of the 0.4 kb fragment reveals amplification of an unrelated region on chromosome 3 (shown in Fig. S6)

The sequence of the amplified fragment from subclone B-5 shows the presence of the canonic AC at the end of the 5’-LTR at the break point with the chromosomal sequence, as expected from the legitimate integration of the provirus. The viral sequence proceeds normally for the first 51 nt of U3 up to the internal forward primer. The PCR results were confirmed using an external primer (F8-R) closer to the detected integration site and the same forward primer. The analysis was extended to all subclones of A-1-26 using the external primer (F8-R) and the same forward primer. Gel analysis of the PCR products showed a band of the expected size (0.26 kb) only in subclone B-5. A band of about 0.4 kb was observed in six other subclones (Fig. 6A). Southern blot analysis of these PCR products showed positive hybridization with an LTR-specific probe only with the 0.26 kb fragment (Fig. 6B). Sequence analysis revealed the 0.4 kb fragment to be the product of the amplification of an unrelated region in chromosome 3 (subclone B-9; Fig. S6).

**Fig. 7:**
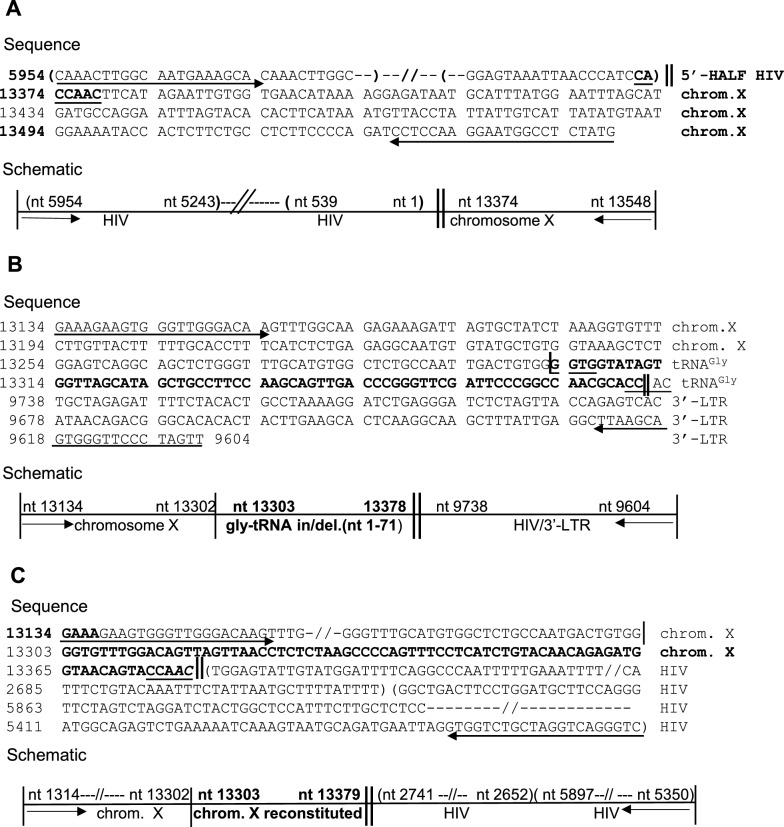
Analysis of sequences flanking the proviral DNA. **A** PCR amplification of a region of the X chromosome of clone A-1-26/B-5 containing the 5’-half of the viral genome (from nt 5954 to nt 1) and flanking chromosomal sequence from primer F8-R. Underlined and in bold are the direct repeats flanking both termini of the provirus and the canonical CA dinucleotide. Schematic: forward primer: 5′-HIV → F8-R (nt 5954 → 5933); reverse primer: F8-R (nt 13348 → 13329). **B** PCR amplification of a region of chromosome X containing the 3′-LTR of HIV integrated in clone A-1–26/B-5 shows the presence of a tRNA^Gly^ flanking the viral DNA. Schematic: forward primer: F8L (nt 13134 → 13154); reverse primer: 3′-U5 (nt 9604 → 9626). **C** PCR amplification of the 3’-half provirus of HIV MN in clone A-1-26/B59. Sequence analysis of a region of chromosome X (nt 13133 → 13379) containing the reconstituted sequence deleted by the insertion of tRNA^Gly^, flanking an HIV fragment translocated from the 5’-end of the provirus (nt 2741 → 2652), joined to a truncated 3’-end (nt 5897 → 5350). Schematic: forward primer: F8-L (nt 13134 → 13154); reverse primer: 3′-HIV → F8-L (5350 → 5369)

*3’- LTR integration site.* Having determined the chromosome sequence at the 5’-end of the provirus, a second external primer, F8-L, was designed to detect the junction of the 3’-LTR and the chromosome. When the forward F8-L primer (from nt 13134) and the reverse primer 3’-U5/MN (from nt 9604) were used, a fragment of 375 nt was amplified. The sequence from the external primer F8-L proceeds for 240 nt up to GT at the end of the 3’-LTR and continues normally for 135 nt into the U5 region up to the internal reverse primer. Surprisingly, 67 of the 240 nucleotides of chromosome X had been replaced by an equal number of nucleotides apparently translocated from chromosome 19 (Fig. 7A). BLAST analysis of this stretch of nucleotides revealed a perfect match of 67 of 72 nt of the tRNA-Gly-TCC1-1 gene (NCBI gene ID: 7197). The first five nucleotides of the 5’-leader strand of the tRNA^Gly^ are missing (Fig. S7A, B). At the 5’-end, the first four nt of the integrated tRNA, GGTG, are also present at the 5’-end of the deleted chromosome X sequence. At the 3’-end, a direct complement of the short GGTG sequence, CCAC, joins the 3’-end of the tRNA^Gly^ to the 3’-LTR, instead of the 5 bases of the staggered integration site, flanking the ends of the provirus (CCAAC). The presence of CCA (the canonical signature added to the 3’-end of mature tRNA molecules) at the end of the 67 nt of the integrated tRNA^Gly^ implies that this integration occurred through an RNA intermediate and is the result of the reverse transcription of a mature tRNA^Gly^ molecule.

*Evidence of provirus instability.* We found evidence of provirus instability while attempting to detect the full-length provirus to confirm its functional integration in subclone B-5 of A-1-26. Using primers F8-L and F8-R external to the LTRs, we obtained a PCR product of 0.415 kb with A-1-26 subclones B-5 and B-9. The respective amplicons showed only chromosomal sequences. The lack of viral sequences is consistent with the tetraploidy of the XY genotype of Jurkat cells [21] or alternatively from complete disintegration of the provirus. The following experiments were aimed at amplifying the two halves of the MN provirus in B-5 using the external primers F8-L (forward) and F8-R (reverse) respectively in combination with internal primers 5’- and 3’- to the unique EcoRI restriction site in the central region of the HIV MN genome.

*PCR amplification of the 5’-half of the provirus.* A 6.1 kb fragment was amplified using the external reverse primer F8-R and the forward internal primer 5’MN → F8-R in the central region of the provirus (from nt 5954), and 700 nt were sequenced at both ends of the fragment. The sequence analysis in the reverse direction (from the external primer F8-R) showed 175 nt of chromosome X and the first 515 nt of the 5’-LTR of the minus strand regularly integrated. The recession of two bases at the 5’-end of the provirus was confirmed and, unexpectedly, a duplication of 44 nt at the 3’-end of this segment was detected. Sequence analysis in the forward direction, from the internal primer 5’MN → F8-R, proceeded regularly from nt 5954 in the direction of the 5’-LTR, up to nt 5217. Identical results were obtained with eight amplicons. Sequences from the two ends of the amplified region of the provirus are shown in Fig. 7B.

*Deletion/translocation events in the 3’-half of the provirus.* The 3’-end of the provirus was amplified using an internal reverse primer (from nt 5356) and the external forward primer F8-L. We analyzed two amplicons containing viral sequences and found that the tRNA^Gly^ had been deleted and replaced by the exact number of nt previously removed by its insertion*.* A large deletion in the provirus including the 3’-LTR and internal rearrangements were detected. Duplication of the bases flanking the 5’-LTR, evidence of the staggered cut created by the HIV integrase, was revealed at the end of the reconstituted chromosome joined to the truncated provirus which includes the last three bases of the reconstituted fragment (ACT). Microhomology may have facilitated the joining of the reconstituted genomic DNA with the viral DNA*.* The viral sequence initiates with a stretch of 90 nt translocated from the 5’-end of the provirus (from nt 2740 to nt 2652) connected to 554 nt of the 3’-half of the viral DNA (from nt 5904 to nt 5356; Fig. 7C). Comparable results were observed with a second amplicon using a different internal reverse primer.

## Discussion

The data presented in this study link the decline of viral expression in an HIV-infected Jurkat line to deletions in viral DNA that occurred early in infection and during cell growth. Large deletions (and hypermutation) in integrated HIV DNA have been reported in PLWH to the extent that only ~ 2% of the proviruses are replication competent [4–6, 11]. It has been suggested that the deletions occur in vivo during the process of reverse transcription; however, the presence of two copies of viral genomes per virion should make it possible for reverse transcriptase (RT) to switch template and complete DNA synthesis [22–25]. Moreover, if the deletions occur in proviral DNA before integration, it is reasonable to expect their presence in the linear or episomal forms, but this has not been reported. Furthermore, complete spontaneous deletion of HIV provirus has not been shown. Precise excision of a retrotransposon has been reported in Drosophila [26], attributed, in that case, to the activity of the retrotransposon IN [27, 28]. In our model, deletions occur after integration. This was best analyzed in a moderate producer clone that progressively lost virus expression over extended time in culture. The decline paralleled that of episomal DNA (Table S4), and the percentage of CD4^+^ cells remained very low throughout the time in culture. Integrated viral DNA was detected only in one of the derived subclones that was, however, positive for episomal and total viral DNA. The co-existence of intact and partially deleted forms of provirus in the same clonal population of the only subclone positive for proviral DNA is suggestive of progressive loss of provirus.

Thus, the loss of proviral DNA was linked to the integration of a reverse transcribed molecule of tRNA^Gly^ next to the provirus. The tRNA^Gly^ abutting the 3’-end of the provirus is not an exact duplicate of the gene present on chromosome 19 (Locus chr19:4724070–4724141). Comparison of the two sequences shows a deletion of five bases at the 5’-end and the addition of four bases (CCAC) at the 3’-end of the integrated form, three of which, CCA, are present at the 3’-end of mature tRNA.

*Proposed model.* We present substantial evidence that the tRNA^Gly^ adjacent to the MN provirus reported in this study is a reverse transcribed mature tRNA. Colicelli and Goff [17] previously observed reverse transcription of a tRNA^Gly^ molecule and described the finding of a molecule of tRNA^Gly^ between the terminal repeats of a cloned circular MMLV DNA. The authors suggested that a spurious primer, a molecule of tRNA^Gly^, was used by the MMLV reverse transcriptase to initiate the synthesis of the minus strand, remained linked to the 3’-end, and was copied after the complete elongation of the plus strand. It was then retained during cyclization of the linear form of viral DNA. Four bases were missing at the 5’-terminus of the inserted tRNA. Large deletions, including the complete LTR, were observed in several other cloned episomal forms [29].

Our observations are strongly analogous to their findings. The proximity of CCA at the end of the integrated tRNA^Gly^ to the 3’-end of the provirus is consistent with the idea that this molecule was utilized as primer and copied during the process of reverse transcription, remaining linked to the 3’-end of the viral DNA. The sequence CCACTG, bridging the integrated tRNA and the 3’-end of the provirus, is the expected result of elongation of the tRNA primer in the U5 region of HIV at the initiation step of reverse transcription. Like the tRNA^Gly^ inserted in the episomal form of MMLV DNA, we report a loss of nucleotides at the 5’-end of the integrated tRNA^Gly^. The fact that 1) the RT of two different viruses would use the same spurious primer for the transcription of their cognate RNA, and 2) that the respective loss of four and five bases from the 5’-end of the leader stem in the two different cases seems more than coincidental and is suggestive of a common mechanism in the reverse transcription of the tRNA^Gly^.

*Alternate hypothesis.* While the proposed model explains how a molecule of tRNA^Gly^ could be retrotranscribed and inserted into a circular form of MMLV DNA [17, 29], some considerations make it unlikely that the integrated form of a tRNA^Gly^ was reverse transcribed by the same mechanism as in our case. First, in the murine model used by Colicelli and Goff, the inability of the MMLV RT to remove the “spurious primer” from the elongated minus strand and to copy it completely at the end of the extension of the plus strand (after the second jump) was suggested to be a consequence of mutations experimentally introduced in the 3’-LTR of the cloned episomal form. However, this assumption is not valid in our specific context, as no alterations are seen in the sequence of the integrated LTRs, and unlike MMLV which can utilize different tRNAs to prime the synthesis of viral DNA [29, 30], HIV is highly selective for the usage of tRNA^Lys^ as its cognate primer for reverse transcription of the HIV genome. The exclusive use and specificity for this primer is supported by numerous studies [31–33].

Besides the different viruses involved, comparison is made between an episomal form and an integrated one, and the assumption should be made that a circular form was obtained in our case as well [17] and that it would be the substrate for the integration of the chimeric structure. For this to occur, the circle at the junction of the 5’-LTR with the tRNA^Gly^ DNA would need to open. However, the lack of a DNA recognition sequence for the HIV integrase at one end of the chimeric form means this enzyme could not be involved in mediating the integration of such forms. It is likely that the integration of provirus and tRNA is the result of two independent events, of which only one, the integration of the provirus, is a legitimate HIV-integrase dependent integration. The latter is supported by the results of our analyses showing recession of two bases at the two ends and the presence of direct repeats flanking the provirus, while the insertion of the tRNA^Gly^ adjacent to the 3’-LTR of MN remains to be explained.

**Fig. 8:**
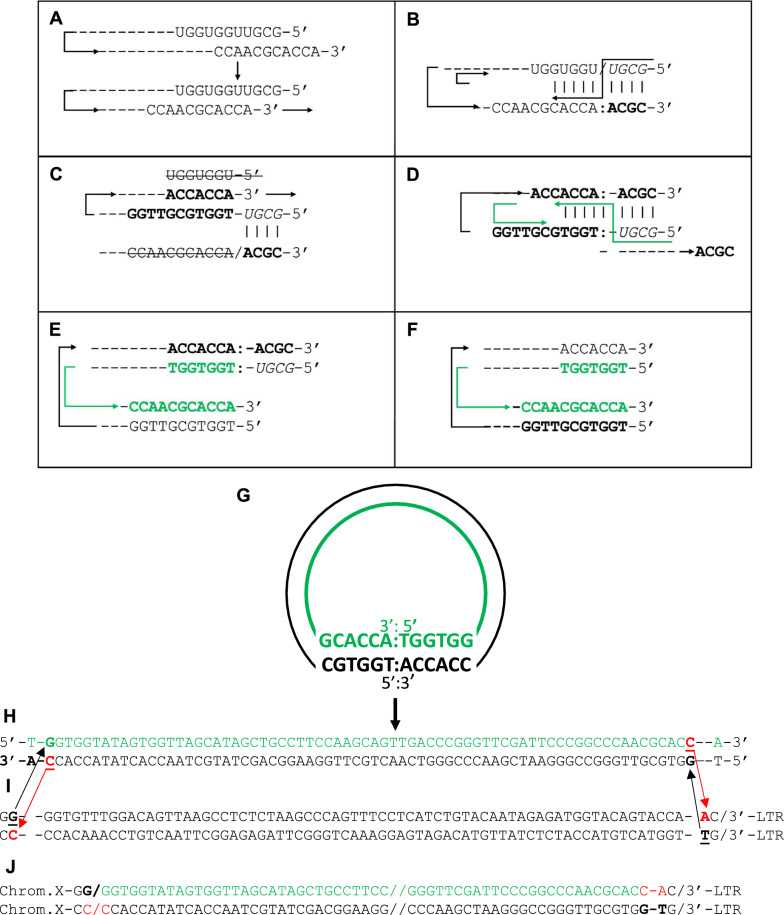
Reverse transcription of tRNA^Gly^ and its insertion on chromosome X adjacent to the 3’-end of the proviral DNA in a clone of a Jurkat cell line infected with HIV MN. **A** Unfolding of the acceptor stem of the tRNA^Gly^ by the RT and binding of the 3’-terminal CCA of the trailer strand to the complementary UGG (nt 5–7) on the leader strand. **B** Extension of the 3’-end generates a short RNA/DNA hybrid (*UGCG*/**ACGC**). A nick at the 3’-end of the RNA (3′-*UGCG*-5′) in this hybrid generates a 3-OH primer for the synthesis of the minus strand. The tRNA^Gly^ is replaced by its complementary DNA, (**C**). **C** The 3’-end of the newly synthesized minus strand (5′-CACCA-3′), annealed to the complementary sequence at the 5’-end (3′-GTGGT-5′), is extended. The four ribonucleotides that primed the synthesis of the minus strand are copied and a short DNA/RNA hybrid is reformed. The four deoxynucleotides (ACGC) that were part of the first hybrid are displaced in (**D**). **D** A nick in the 3’-end of the RNA (3′-*UGCG*-5′) in the new DNA/RNA short hybrid generates a 3’-OH primer for the synthesis of the plus strand. The RNA primer is extended on a DNA template and removed. **E**, **F** The synthesis of the double-strand DNA is completed. **G** Upon removal of the ribonucleotides, the short overhang DNA, ACGC, is deleted to allow ligation of double-strand DNA. The linear form of the retrotranscribed tRNA^Gly^ lacks the first four bases of the leader strand and shows a 5 base inverted repeat at the two termini. Compared with the sequence integrated on chromosome X (**H**–**J**), an extra base is observed at each end

These considerations provide a rationale for proposing an alternative hypothesis. To this end, we also propose an alternative model for the reverse transcription of tRNA^Gly^ that does not involve its usage as primer for the synthesis of viral DNA (Fig. 8A-F). Without the mediation of the cognate factors required for selection and annealing of a specific primer to the primer binding site (PBS), we hypothesize that HIV RT or a cellular RT binds to and unfolds the acceptor stem of the non-cognate tRNA^Gly^ and directs the annealing of the free 5′-CCA-3′ at the end of the trailer strand with the UGG in nt position 5–7 of the leader strand and following bases similar to the binding of the 3’-end of tRNAs (CCA) to the UGG sequence of the retroviral PBS (Fig. 8A). The UGG in nt positions 5–7 is present only in the acceptor stem of tRNA^Gly^ and tRNA^Glu^ adjacent to a second UGG [34]. A short sequence (six nt) at the 5’-end of the PBS is sufficient for recognition and priming by the RT, as shown in the binding of tRNA^Lys^ to the HIV genome [35]. Unfolding the acceptor stem of the tRNA^Gly^ with consequent slippage of the trailer on the annealed leader strand allows for the formation of a primer for the synthesis of the complementary DNA strands (illustrated in Fig. 8).

The end-product of the process described in the proposed model, a linear double stranded tRNA DNA lacking the first four bases of the leader strand at the 5’-end as in the reported murine model [17], is the immediate precursor of the integrated one. It shows two inverted repeats at the termini (TGGTG/CACCA) and an extra base at the two ends when compared to the integrated form, likely removed by the nuclease involved in the deletion/insertion process (Fig. 8F). No direct repeats at the termini of the inserted sequence are observed. The integrated tRNA^Gly^ shows homology at the two ends with the genomic DNA at the break points (Fig. 8G). This is reminiscent of the IN-independent mechanism of integration of Tf1-INfs mediated by the Rad52-dependent single-strand annealing (SSA) pathway described by Li et al. [36].

This model of reverse transcription of tRNA^Gly^ provides an explanation for the loss of exactly four bases from the 5’-end of this molecule observed in both models. In addition, it seems appropriate to observe that insertion of the double stranded tRNA DNA with unintegrated proviral DNA would produce a chimeric form (Fig. S8) similar to the circular form described by Colicelli and Goff [17]. The ability of the RT to unwind the acceptor stem of primer tRNA is supported by in vitro studies on the interaction of the AMV RT and its tRNA primer using enzymatic and chemical probes [37]. Unwinding of the acceptor stem of tRNA^Lys^ by the HIV RT was suggested by foot-printing analysis demonstrating that the digestion of this region of the tRNA occurred only in the presence of the viral enzyme [38, 39]. However, the result was disputed in another study and attributed to excessive nuclease digestion [40]. Support for the idea that binding of RT to tRNA^Lys^ resulted in opening the acceptor stem was subsequently provided by Oude Essink et al*.* [41] who demonstrated binding of PBS-mimicking nucleotides to a preformed tRNA-RT complex, but not to tRNA alone.

Reverse transcription of tRNA or tRNA-like molecules has been described in different systems. Lambowitz and colleagues documented the presence of a reverse transcribed mitochondrial tRNA between the terminal ends of the mitochondrial retroplasmids of two Neurospora strains [42, 43]. The reverse transcription of the mitochondrial plasmid RNA is self-primed by an encoded reverse transcriptase that recognizes the 3′-CCA of a tRNA structure present at the 3’-end of the plasmid transcript. Wittig and Wittig reported in vitro reverse transcription of an oligo-A extended tRNA^Gly^, primed by oligo dT, by the Klenow fragment of the E. coli DNA polymerase I [44]. Most relevant to our study, Colicelli and Goff provided the first report of a reverse transcribed tRNA^Gly^ in a murine system [17]. Thus, the ability to recognize, unfold, and transcribe tRNA structures is in the repertoire of reverse transcriptase enzymes, and different strategies are involved in the transcription of these molecules.

Our data are consistent with the idea that the molecules of tRNA^Gly^ and proviral DNA integrated independently at the same site. We suggest that integration of the tRNA^Gly^ followed that of the provirus, which we plan to further examine by extending the analysis to other clones of the infected line at different timepoints. At present, we propose that the insertion of this tRNA introduced genomic instability resulting in the reported deletions as tRNA genes can induce genomic collision between the transcription and replication machinery, resulting in double strand breaks [45]. Particularly intriguing is the presence of a structure (5′-TGGGGTGG-3′) resembling the Chi sequence 5′-GCTGGTGG-3′ at the breakpoint between the 5’-end of inserted tRNA^Gly^ and the genomic DNA. Although RecBCD is not present in mammalian cells, this sequence could have functioned as a signal for DNA repair by a similar enzyme [46].

tRNAs function as primers for the RT activity of retrovirus and retrotransposons, and cells use specialized machinery to silence both epigenetically [47]. In addition, tRNA fragments and halves are utilized by cells in the post-transcriptional control of retrotransposons [48], and tRNAs could similarly be utilized to inactivate retroviruses. Our results imply that this is indeed the case, and while virus utilizes tRNA for its replication, infected cells can utilize the same tool to target viral DNA and irreversibly inactivate the virus.

## Ethics approval and consent to participate

Not applicable.

## Consent for publication

Not applicable.

## Supplementary Information

Supplementary Material 1.

## Data Availability

Data is provided within the manuscript or supplementary information files.
