# Supplementary material for: t-RNA mediates provirus deletion in HIV-infected cells
Source: Retrovirology. 2025 Jul 1;22:11. doi: 10.1186/s12977-025-00667-0 (PMC12220202; doi:10.1186/s12977-025-00667-0)
Supplement: Supplementary file 1 — Supplementary Material 1. [file 12977_2025_667_MOESM1_ESM.pdf]

Figure S1

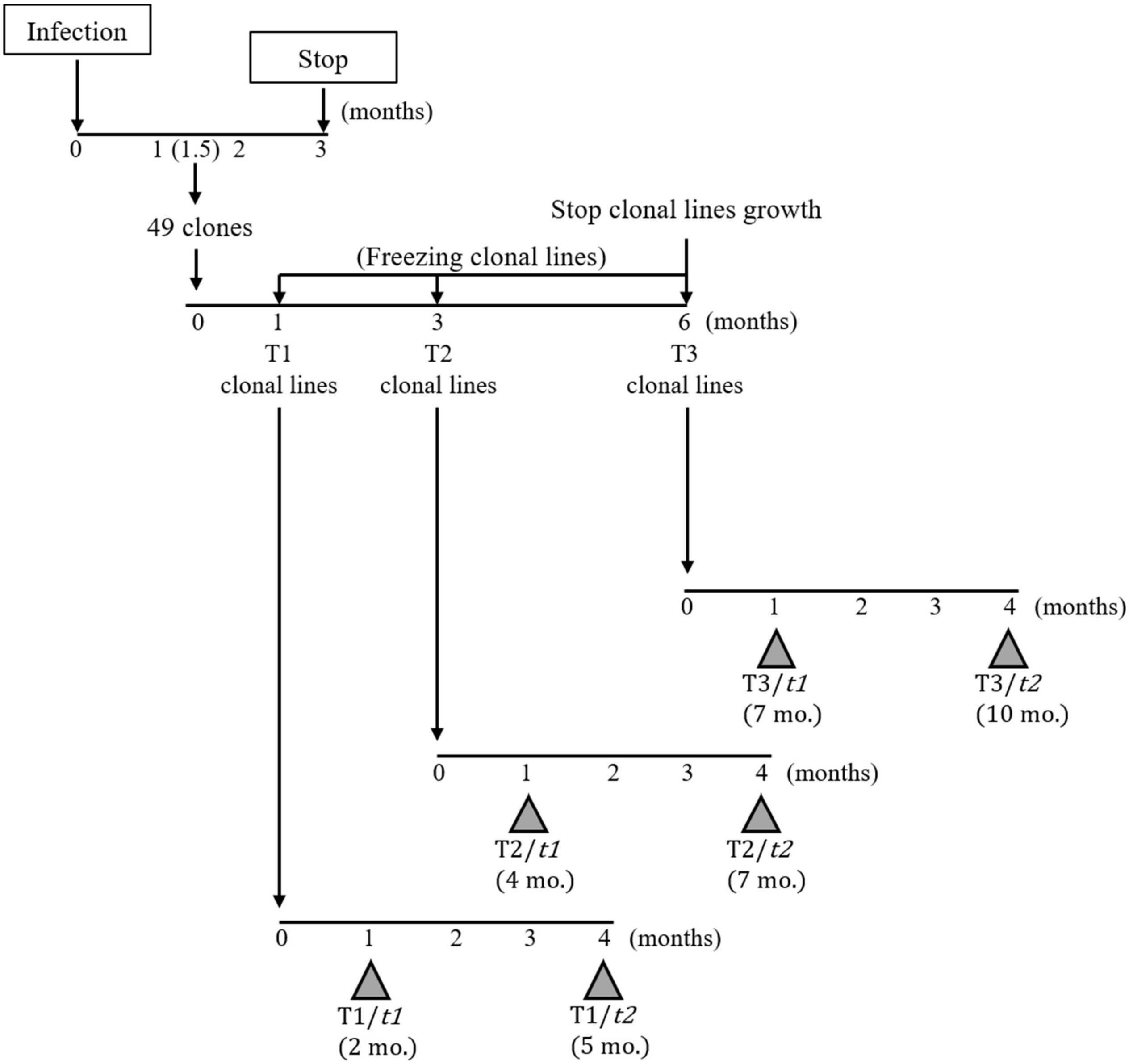

Fig. S1. Isolation of clones from a Jurkat line infected with an early isolate of HIV MN. Forty-nine clones were isolated from J20/MN by limiting dilution, maintained in culture for six months, and frozen after 1 (T1), 3 (T2), or 6 (T3) months of growth from single cell. Samples were thawed and analyzed after an additional 1 month (*t1*) and 4 months (*t2*) of continuous growth.

Figure S2

A

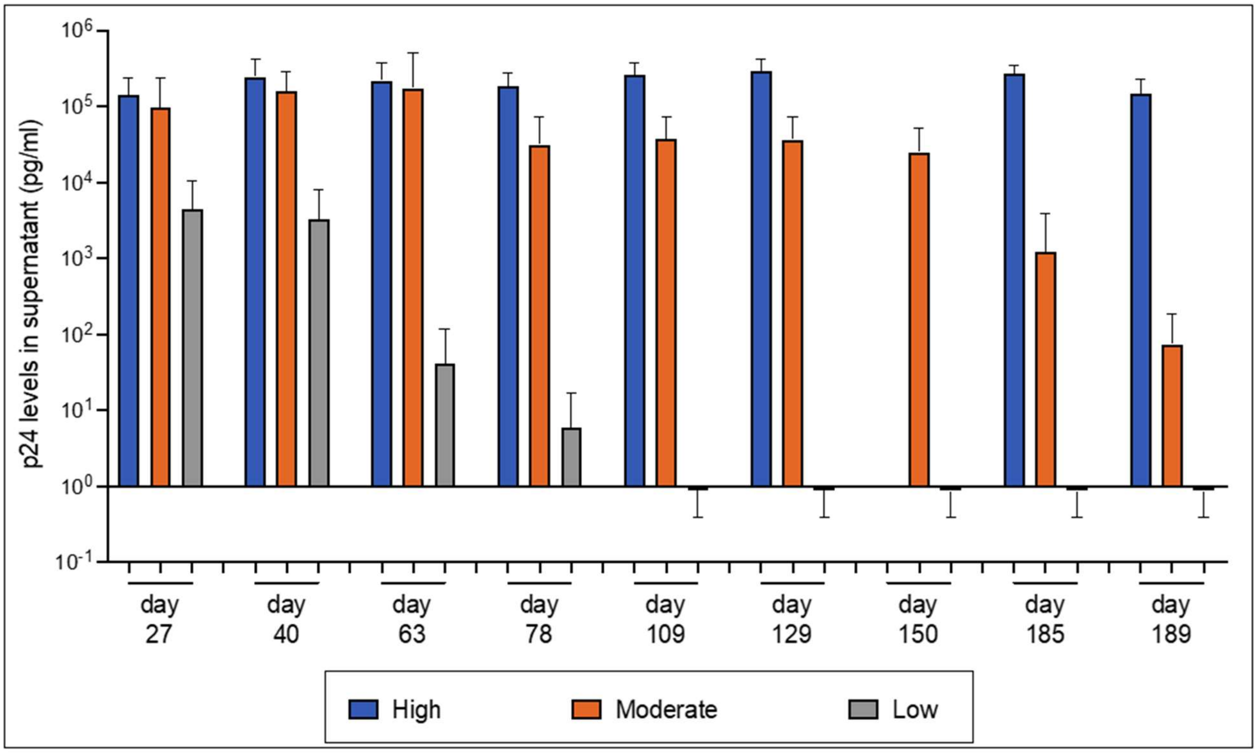

p24 levels in supernatant (pg/ml)
10^6
10^5
10^4
10^3
10^2
10^1
10^0
10^-1
day 27
day 40
day 63
day 78
day 109
day 129
day 150
day 185
day 189
High
Moderate
Low

B

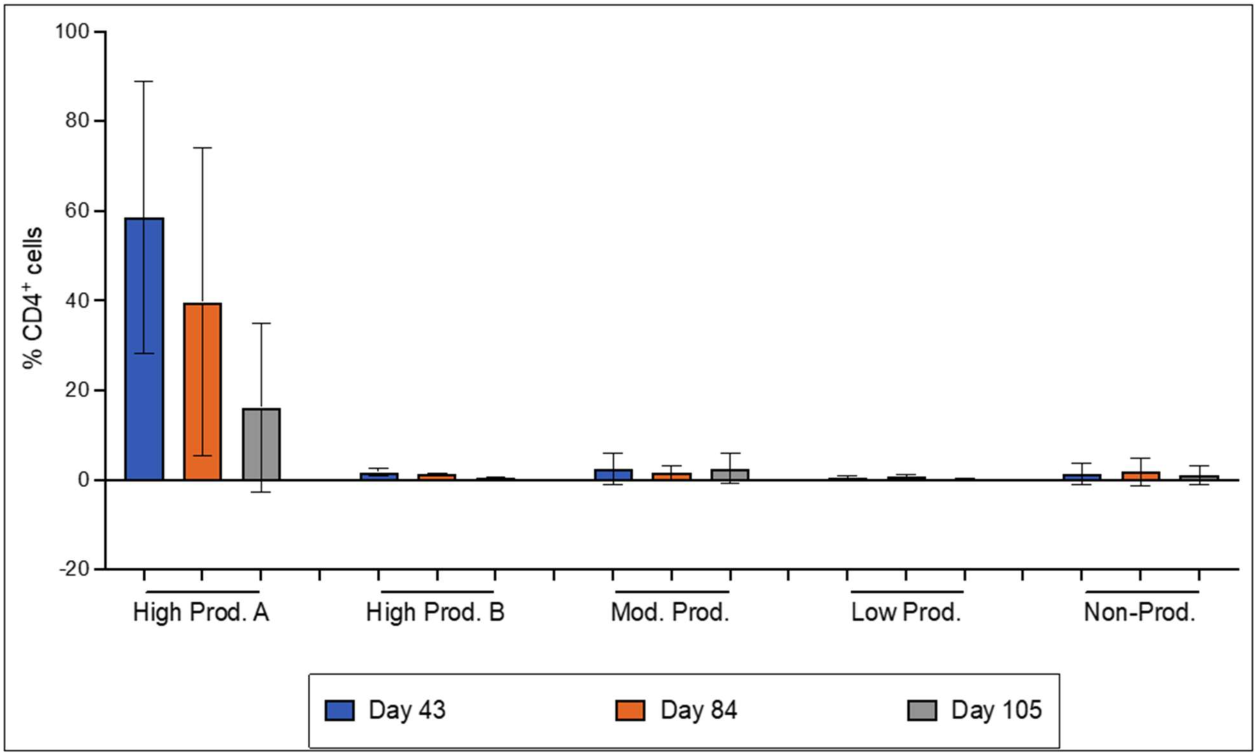

% CD4+ cells
100
80
60
40
20
0
-20
High Prod. A
High Prod. B
Mod. Prod.
Low Prod.
Non-Prod.
Day 43
Day 84
Day 105

C

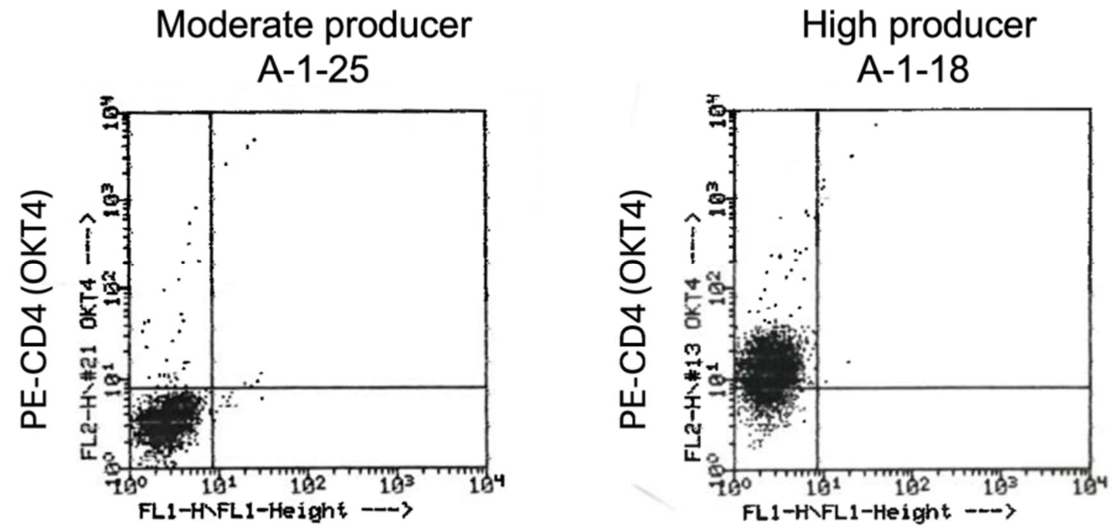

Fig. S2. Expression of p24 (pg/ml) and % CD4$^+$ cells in high, moderate, and low producer clones of J20/MN. (A) Average value of p24 released by clones of J20/MN measured as a function of the time in culture as shown in Table S2. (B) Average percent CD4$^+$ cells in clones of J20/MN measured as a function of time in culture; data is reported in Table S3. (C) Representative flow cytometry gating strategy to assess the frequency of CD4$^+$ cells in the clones J20/MN. One moderate (clone A-1-25) and one high (A-1-18) producer are reported. Gating strategy: FL2-PE-CD4 (OKT4) vs FL1-FITC-unstained.

Figure S3

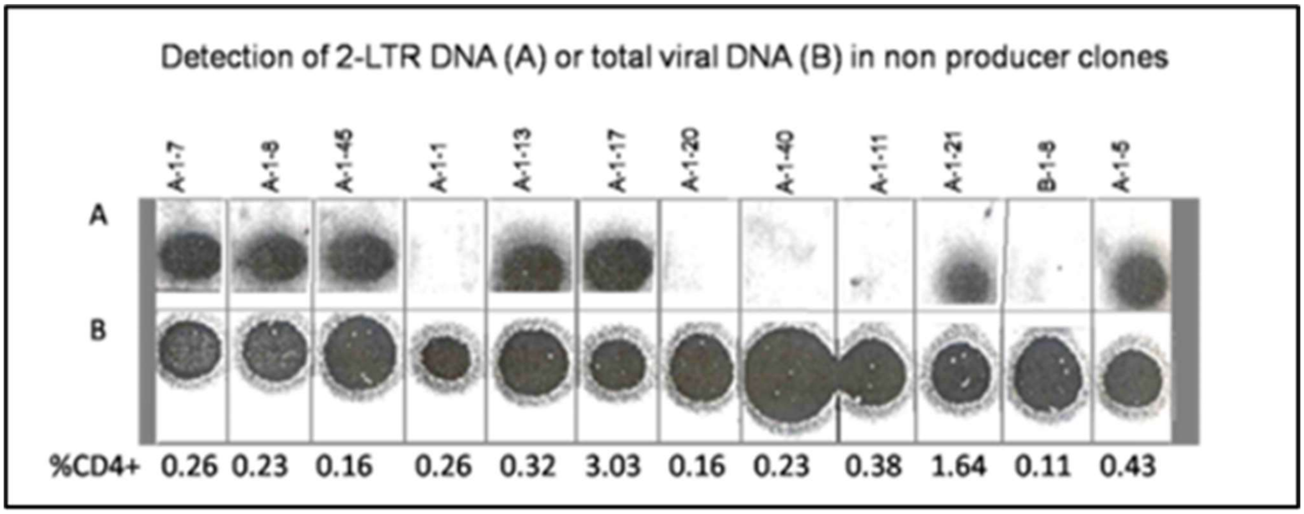

Fig. S3. PCR analysis of nonproducer clones. (A, B) PCR analysis detects the presence of (A) 2-LTR and (B) total DNA in nonproducer clones of J20/MN (12 clones are shown). Provirus was not detectable by Southern blot analysis. Two selected clones (A-1-20 and A-1-40) were provirus negative by Alu PCR.

Figure S4

A

```
A-1-20   1        ACTGCACTCCAGCCTGGGCGACAGAATGGGAGAGTAAAGAAAGAGAGAAAGAAAGACAGA   60
                  |||||||||||||||||| ||||||||  |||||||||||||||||||||||||||||||
Chrom.2  118663   ACTGCACTCCAGCCTGGGTGACAGAAT--GAGAGTAAAGAAAGAGAGAAAGAAAGACAGA   118720

A-1-20   61       GAGAGAGAGAAAGAAAGGAAGGAAGGAAGGAAGGAAGACCTGAGCAAGTGCGCCTG   120
                  |||||||||||||||||||||||||||||||| |||||||||||||||||||||||
Chrom.2  118721   GAGAGAGAGAAAGAAAGGAAGGAAGGAAGGAA-GAAGACCTGAGCAAGTGCGCCTG   118779

A-1-20   121      CTCTGTCTCACCACGTGATGCTTCCCGCTGTGTCATGGCGCAGCAGGTGGGCCCTCACCA   180
                  ||||||||||||||||||||| ||||||||||||||||||||||||||||||||||||||
Chrom.2  118780   CTCTGTCTCACCACGTGATGCCTCCCGCTGTGTCATGGCGCAGCAGGTGGGCCCTCACCA   118839

A-1-20   181      GAAGCAGGTGCCAATGCTCTTGGACTTCCTAGCCTCCAGGAAAATGTGCTGAATAAATAT   240
                  ||||||||||||||||||||||||||||||||||||||||||||||||||||||||||||
Chrom.2  118840   GAAGCAGGTGCCAATGCTCTTGGACTTCCTAGCCTCCAGGAAAATGTGCTGAATAAATAT   118899

A-1-20   241      CTATTCTTTATAAATTGTGCAGTCTGTGGTGTTCAGTTATAGCACTAACACACGGACAAG   300
                  ||||||||||||||||||||||||||||||||||||||||||||||||||||||||||||
Chrom.2  118900   CTATTCTTTATAAATTGTGCAGTCTGTGGTGTTCAGTTATAGCACTAACACACGGACAAG   118959

A-1-20   301      ACATCCT 307
                  |||||||
Chrom.2  118960   ACATCCT 118966
```

B

Sequence of the ACH2 DNA fragment amplified by ALU PCR

```
 1 TCCACAGATC AAGGATATCT TGTCTTCTTT GGGAGTGAAT TAGCCCTTCC AAATACGGTT 60
61 GAGTCCAGAA AATTGCCAGA TGAGGCGGCT CACGCTGTAA TCCCAGCA            108
```

Human immunodeficiency virus type 1 (HXB2) ID: K03455.1

```
Ach2/clone 1 TCCACAGATCAAGGATATCTTGTCTTCTTTGGGAGTGAATTAGCCCTTCCA 51
             ||||||||||||||||||||||||||| |||||||||||||||||||||||
HXB2      51 TCCACAGATCAAGGATATCTTGTCTTCGTTGGGAGTGAATTAGCCCTTCCA 1
```

Pan troglodytes BAC clone CH251-561B24 from chromosome 8 ID: AC195294.3

```
Ach2/clone   52 AATACGGTTGAGTCCAGAAAATTGCCAGATGAGGCGGCTCAC-GCTGTAATCCCAGCA 108
                 |||||||||||||||||||||||||||||||||| ||||||  ||| ||||||||||
Chrom.8  191594 AATACGGTTGAGTCCAGAAAATTGCCAGATGAGGCAGCTCACACCTGCAATCCCAGCA 191537
```

C

Sequence of the ACH2 DNA fragment amplified by ALU PCR

```
  1 TGTGGGTTAC AGGCGTGAGC CACCGTGCCC GGCCAGTTCT ATGAGTTTTG ATGAATGTGT 61
 61 CCTGCTTCTT CCCAGGCTCC ACCATCTCTT CCCCTAAAGG CAACCACCAT TCTGATTTCT 121
121 CTCACCAAAG ACTGGAAAAA TATCCCAATA CCTGGATGTA TGCCTCACGT GTCCCGCTGG 181
181 CCCCAGCGCC CAGGTATAGT TAGTTACTGC TGTGCCTGGC TTCACTCTTG GAAGGGCTAA 241
241 TTCACTCCTA AAGAAGACAA GATATCCTTG ATCTGTGGA  279
```

Human DNA sequence from clone RP11-395P17 on chromosome 9 ID: AL590708.18

```
Ach2/clone     3 TGGG-TTACAGGCGTGAGCCACCGTGCCCGGCCAGTTCTATGAGTTTTGATGAATGTGTC 6
                 |||| |||||| ||||||||||||||||||||||||||||||||||||||||||||||||
Chrom.9    13178 TGGGATTACAGACGTGAGCCACCGTGCCCGGCCAGTTCTATGAGTTTTGATGAATGTGTC 13119

Ach2/clone    62 CTGCTTCTTCCCAGGCTCCACCATCTCTTCCCCTAAAGGCAACCACCATTCTGATTTCTC 121
                 ||||||||||||||||||||||||||||||||||||||||||||||||||||||||||||
Chrom.9    13118 CTGCTTCTTCCCAGGCTCCACCATCTCTTCCCCTAAAGGCAACCACCATTCTGATTTCTC 13059

Ach2/clone   122 TCACCAAAGACTGGAAAAATATCCCAATACCTGGATGTATGCCTCACGTGTCCCGCTGGC 181
                 ||||||||||||||||||||||||||||||||||||||||||||||||||||||||||||
Chrom.9    13058 TCACCAAAGACTGGAAAAATATCCCAATACCTGGATGTATGCCTCACGTGTCCCGCTGGC 12999

Ach2/clone   182 CCCAGCGCCCAGGTATAGTTAGTTACTGCTGTGCCTGGCTTCACTCT 228
                 |||||| ||||||||||||||||||||||||||||||||||||||||
Chrom.9    12998 CCCAGCACCCAGGTATAGTTAGTTACTGCTGTGCCTGGCTTCACTCT 12952
```

Human immunodeficiency virus type 1 (HXB2) ID: K03455.1

```
Ach2/clone   229 TGGAAGGGCTAATTCACTCCTAAAGAAGACAAGATATCCTTGATCTGTGGA 279
                 |||||||||||||||||||| || |||||||||||||||||||||||||||
HXB2           1 TGGAAGGGCTAATTCACTCCCAACGAAGACAAGATATCCTTGATCTGTGGA  51
```

Fig. S4. Sequence blast analysis. (A) Primers used for the detection of provirus in clones of J20/MN amplify an unrelated region on chromosome 2 in a random chosen nonproducer clone (A-1-20) (Sequence ID: AC093740.2). Forward primer (Alu, nt 240/261): 5'-ACTGCACTCCAGCCTGGGCGAC-3'. Reverse primer (5'-MNLTR, nt 51/28): 5'-CCCACAGATCAAGGATGTCTTGTC-3'. (B, C) Blast analysis of the sequence of a fragment of (B) chromosomes 8 and (C) chromosome 9 of the clonal line ACH-2 containing an integration site of HIV. Forward primer (5'-MNLTR, nt 51/28). Reverse primer: Alu reverse primer: 5'-TGCTGGGATTACAGGCGTGAG-3'.

Figure S5

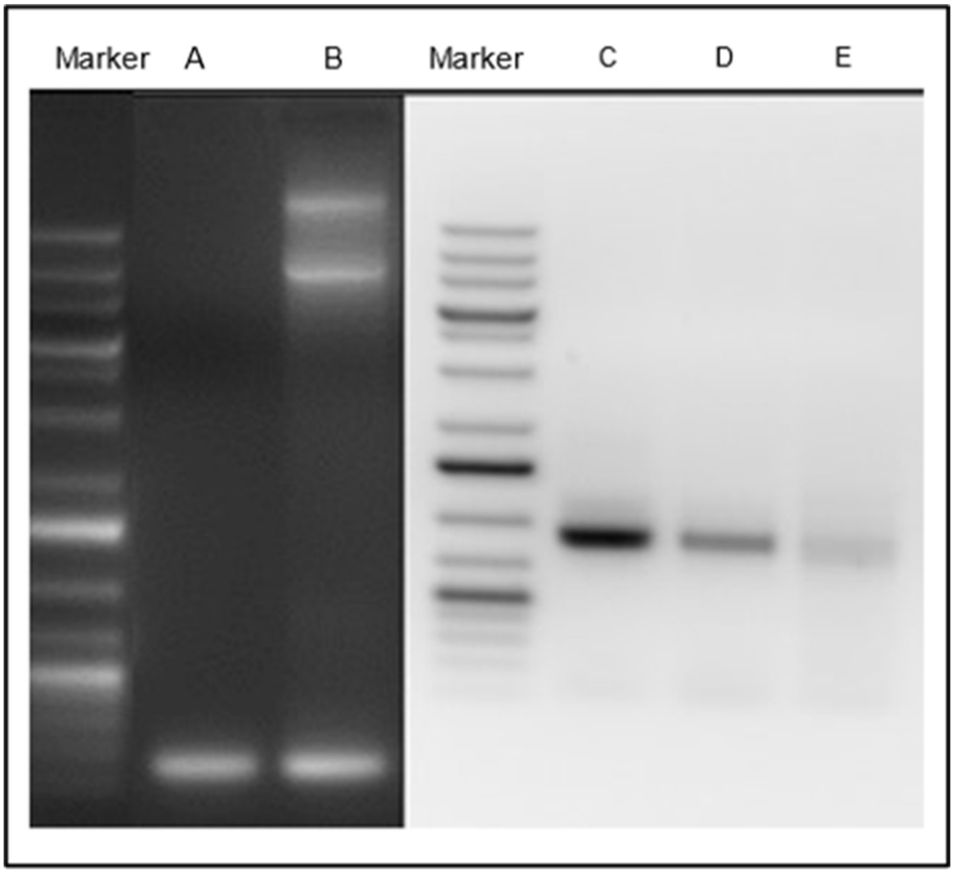

Fig. S5. Infectivity of virus released from clone B-5. To demonstrate the infectivity of the virus released by the B-5 clone, $1X10^6$ cells of an early passage of the clonal line were co-cultivated with an equal number from the $CD4^+$ J20 T cell line. After 24 h the supernatant was removed and used to infect a second batch of J20 cells. Viral DNA was detected by PCR analysis of the DNA extracted 3 days after infection. (A) Amplification of total DNA in the infected J20 cells and (B) in pHXB2. (C–E) Amplification of 1-LTR episomal form [20] present in the DNA of the infected J20 DNA extracted at 3 different time points. A 1kb Plus DNA ladder was used as marker.

Figure S6

```
B-9                            CCCACAGATCAAGGATGTCTTGTCCTTGGAGAGGTCCGGT   51
Chr.3   AGGTTACATAGGTGTAATCTGGATGTCAGCTGAGATGTCTTGTCCTTGGAGAGGTCCGGT   33594651
B-9     TCTCTCCCAGCTTCTCTGGGTATTTTGGAGGAGGTGTACATTTTCCCACTTCCCTCTTAG   111
Chr.3   TCTCTCCCAGCTTCTCTGGGTATTTTGGAGGAGGTGTACATTTTCCCACTTCCCTCTTAG   33594711
B-9     CTCTGAGCTTCGGTTGTAACTGAGGTGGTTCCATTTTTGGATTGTCTTTCTGAGCCATAT   171
Chr.3   CTCTGAGCTTCGGTTGTAACTGAGGTGGTTCCATTTTTGGATTGTCTTTCTGAGCCATAT   33594771
B-9     GGGCTGTTTCTGTTCTGCCTTCAATTGCTCCTAATGTATGACACATTCTCTTTTCCTTAC   231
Chr.3   GGGCTGTTTCTGTTCTGCCTTCAATTGCTCCTAATGTATGACACATTCTCTTTTCCTTAC   33594831
B-9     ACCAGGCAGGTGGGTGTCTTGCAAAACTGACTGATGATTTAAGATTGAGGAGGGCTATGT   291
Chr.3   ACCAGGCAGGTGGGTGTCTTGCAAAACTGACTGATGATTTAAGATTGAGGAGGGCTATGT   33594897
B-9     CACAGTATCTCCCAGCAGACAGCCCCAAGGTAAACAGAAGACATCCCCTGTCCTCAAGGC   351
Chr.3   CACAGTATCTCCCAGCAGACAGCCCCAAGGTAAACAGAAGACATCCCCTGTCCTCAAGGC   33594957
B-9     TCCTTTGGCCTCCCCAGGAAGGGCCCCAGACACTCACACACTCTCTTTCCTCCAAGGAAT   411
Chr.3   TCCTTTGGCCTCCCCAGGAAGGGCCCCAGACACTCACACACTCTCTTTCCTCCAAGGAAT   33595017
B-9     GGCCTCTATG--------------------------------------------------   421
Chr.3   GGCCAGGTCTCTGTTTTCTGGGACCCTCAGCTTTTGGTTCCCCCAACTTCATTCCGCCAC   33595077


5'-CCCACAGATCAAGGATGTCTTGTC-3'  (Primer MH536/MN)
5'-GTATCTCCGGTAAGGAACC-3'       (Primer F8-R)
```

Fig. S6. Amplification of a region of chromosome 3. PCR primers 5'-U3/MN and F8-R, which detect the integration site of the HIV provirus in one out of eight subclones (B-5) of A-1-26, amplify an unrelated region on chromosome 3 in the other subclones of A-1-26.

Figure S7

A

Sequences.

1) 5′-GCGTT-GGTGGTATAGTGGTTAGCATAGCTGCCTTCCAAGCAGTTGACCCGGGTTCGATTCCCGGCCAACGCA-3′
2) 5′-(-----)-**GGTG**GTATAGTGGTTAGCATAGCTGCCTTCCAAGCAGTTGACCCGGGTTCGATTCCCGGCCAACGCA**CCAC**-3′
3) 5′- -GGTGTTTGGACAGTTAGTTAACCTCTCTAAGCCCCAGTTTCCTCATCTGTACAACAGAGATGGTAACAGTA=
-CCAAC-3′

B

Sequence alignment.

```
2) 5′-GGTGGTATAGTGGTTAGCATAGCTGCCTTCCAAGCAGTTGACCCGGGTTCGATTCCCGGCCAACGCACCAC-3′
3) 5′-GGTGTTTGGACAGTTAGTTAACCTCTCTAAGCCCCAGTTTCCTCATCTGTACAACAGAGATGGTAACAGTACCAAC-3′

2) 1====GGTGGTATAGT-GGTTAGCATAGCTGCCTTCCAAGCAGTTGACCCGGGTTCGATTCCCGGCCAACGCACCAC-71
        | | | | | |  |||| | |  ||  |  |||      | | | |      |     ||  |  | ||||
3) 1-GGTGTTTGGACAGTTAGTTAACCTCTCTAAGCCCCAGTTTCCTCATCTGTACAACAGAGATGGTAACAGTACCAAC-76
```

1) Sequence of the tRNA^{Gly} (TCC) gene, ID: CP034522.1
2) Sequence of retrotranscribed tRNA^{Gly} (TCC) integrated on chromosome X, adjacent to the 3′end of HIV.
3) Sequence of chromosome X deleted by the insertion of the retrotranscribed tRNA^{Gly}.

Fig. S7. Sequence analyses. (A) Sequence of (1) the tRNA^{Gly} gene (NCBI gene ID: 7197) and (2) the reverse transcribed tRNA^{Gly} inserted in chromosome X adjacent to the HIV provirus in clone of A-1-26. Sequence comparison shows that 5 bases present at the 5'-end of the tRNA^{Gly} gene (1) are missing in the sequence of the integrated tRNA^{Gly}. Four bases complementary to the first four bases of the inserted tRNA connect this sequence with the 3'-LTR of the HIV provirus. (B) Comparison between the sequence of the tRNA^{Gly} inserted on chromosome clone A-1-26 and the genomic sequence deleted by its insertion.

Figure S8

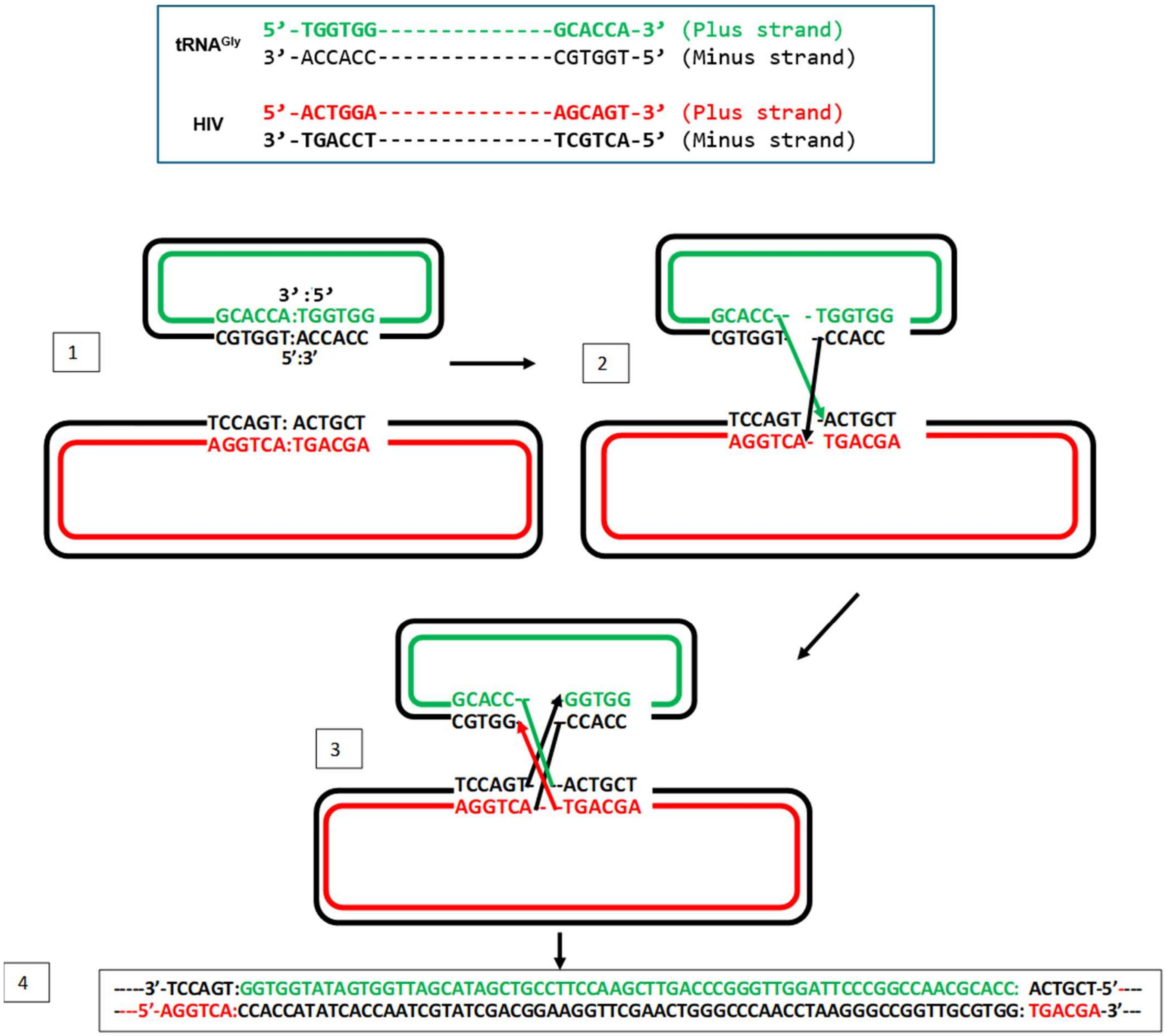

Fig. S8. Insertion of a reverse transcribed tRNA^{Gly} between the terminal repeats of a circular HIV DNA. One terminal base is removed from the 3' ends of the double strand reverse transcribed tRNA^{Gly}. Concerted Insertion of the processed ends occurs following nucleophilic attacks on a single site at the junction of the two LTR of an episomal form of HIV, facilitated by the two bases homology at the respective termini.

Table S1

High producers	Moderate producers	Low producers	Non-producers
A-1-18	B-1-7	A-1-24	A-1-7
A-1-34	A-1-39	A-1-27	A-1-8
B-2-2	A-1-42	B-1-2	A-1-32
A-1-2	A-1-41	A-1-10	A-1-45
A-1-29	A-1-26	A-1-22	A-1-1
A-1-38	A-1-33	A-1-30	A-1-13
A-1-43		A-1-19	A-1-17
A-1-31		A-1-12	A-1-20
B-1-3		A-1-44	A-1-40
B-1-6			B-1-1
A-1-3			A-1-41
A-1-14			A-1-36
B-1-5			A-1-11
			A-1-21
			B-1-8
			A-1-4
			A-5
			A-1-9
			A-1-23
			B-2-1

Table S1. Clones of J20 infected with HIV MN. Based on the amount and persistence of viral antigen released into the culture supernatant over six months of continuous growth, clonal lines were characterized as high (13), moderate (7), and low (9) producers, or nonproducers (20).

Table S2

Expression of p24 (pg/ml) in the supernatant of J20/MN clones as a function of time in culture									
**High Producers**	day 27	day 40	day 63	day 78	day 109	day 129		day 185	day 189
A-1-18*	202342	180000	171000	156000	187900	169000		209000	35000
A-1-43*	138752	187000	336000	257000	342000	361000		291000	207000
B-1-3*	226918	353000	431000	164000	352000	404000		315000	154000
A-1-3				293000	360000	447000		366000	98000
A-1-34*	220364	600000	420000	278000	444000	531000		410000	287000
B-2-2*	115097	354000	301000	144000	129000	204000		169000	117000
A-1-2*	49203	344000	94000	363000	344000	271000		328000	73000
A-1-29*	23765	64716	57530	89240	178000	313000		310000	212000
A-1-38	162406	424000	442777	251000					
A-1-31*	281804	118000	84000	138000	284000	278000		262000	166000
B-1-6*	18982	29000	86000	60000	85600	119000		173000	74000
A-1-14				315000	357000	398000		465000	202000
B-1-5				120000	465000	349000		584000	183000
Sum	1277227	2229716	1980530	1649240	2346500	2650000		2467000	1325000
Average	141914.1	247746.2	220058.9	183248.9	260722.2	294444.4		274111.1	147222.2
S.d.	96822.51	181031.6	152341.5	97118.73	120504.2	126615.7		79272.39	80687.33
**Mod. Producers**	day 27	day 40	day 63	day 78	day 109	day 129	day 150	day185	day 189
A-1-16*	500	122000	64000	42000	50000	84000	39500	97	81
A-1-39*	342000	286000	20400	10900	16600	21800	15600	6000	269
A-1-25*	34800	84000	14400	5400	270	28	0	0	0
A-1-42*	176	138	324	114	23800	62900	65500	0	0
B-1-7	86700	164800	91300	93200					
A-1-26*	109000	302000	780000	100000	95500	15900	6000	4.4	19.5
A-1-33	61388	180000	195000	10100					
Sum	486476	794138	879124	158414	186170	184628	126600	6101.4	369.5
Average	97295.2	158827.6	175824.8	31682.8	37234	36925.6	25320	1220.28	73.9
S.d.	143809.4	131157.3	338580.8	41512	37183.56	35081.91	27038.62	2672.266	114.0134
**Low Producers**	day 27	day 40	day 63	day 78	day 109	day 129	day 150	day185	day 189
A-1-24	3141	380	88						
A-1-27*	239	15	4	0	0	0	0	0	0
B-1-2									
A-1-10*	81	1.1	3	1.1	1.1	1.1	1.1	1.1	1.1
A-1-22	35968	113	222						
A-1-30*	10547	5920	24	1.1	1.1	1.1	1.1	1.1	1.1
A-1-19*	65	1.1	1.1	1.1	1.1	1.1	1.1	1.1	1.1
A-1-12*	11712	10259	177	26	1.1	1.1	1.1	1.1	1.1
A-1-44	104	71	257	280					
Sum	22644	16196.2	209.1	29.3	4.4	4.4	4.4	4.4	4.4
Average	4528.8	3239.24	41.82	5.86	0.88	0.88	0.88	0.88	0.88
S.d.	6040.031	4685.892	76.13581	11.26867	0.491935	0.491935	0.491935	0.491935	0.491935
* Sum, Average, and Standard Deviation values were calculated for selected clones									

Table S2. Expression of p24 (pg/ml) in the supernatant of J20/MN clones as a function of time in culture. Expression was measured in high, moderate, and low producer clones of J20/MN measured over six months of continuous growth.

Table S3

Percentage (%) of $CD4^+$ J20/MN cells as a function of time in culture.

**High Producers (a)**	day 43	day 84	day 105
A-1-18*	92	79	38
A-1-43*	51	15.7	5.4
B-1-3*	33	24.4	5.2
A-1-3		21.26	5.4
Sum	176	119.1	48.6
Average	58.7	39.7	16.2
S.d.	30.24	34.32	18.88
**High Producers (b)**	day 43	day 84	day 105
A-1-34*	2.88	1.41	0.45
B-2-2*	1.91	1.54	0.3
A-1-2*	1.34	1.24	0.3
A-1-29*	2.27	1.66	0.88
A-1-31*	1.21	1.15	0.7
B-1-6*	1	0.85	0.2
A-1-14		2.14	0.4
B-1-5		6.97	1.6
Sum	10.61	7.85	2.83
Average	1.77	1.3	0.472
S.d.	0.72	0.29	0.26
**Mod. Producers**	day 43	day 84	day 105
A-1-16*	0.87	4.4	8.3
A-1-39*	0.78	0.69	2.184
A-1-25*	0.22	1.01	0.22
A-1-41*	2.37	1.05	0.5
B-1-7	2.42	2.08	
A-1-26*	8.6	1.46	1.2
A-1-33	0.69	1.3	
Sum	12.84	8.61	12.4
Average	2.57	1.722	2.5
S.d.	3.46	1.52	3.34

* Sum, Average, and Standard Deviation values were calculated for selected clones

**Low Producers**	day 43	day 84	day 105
A-1-44	4.33		
A-1-22	1.25		
A-1-27*	0.9	0.98	0.22
B-1-2	0.78	0.87	
A-1-10*	0.62	0.33	0.18
A-1-30*	0.4	1.11	0.18
A-1-12*	0.22	0.3	0.6
A-1-24	0.17		
Sum	2.14	2.72	1.18
Average	0.535	0.68	0.295
S.d.	0.29	0.425	0.2
**Non-Producers**	day 43	day 84	day 105
A-1-7	0.87	0.41	
A-1-8*	0.23	0.8	0.26
A-1-32*	0.35	1.01	0.14
A-1-45*	0.42	0.99	0.06
A-1-1*	0.62	0.33	0.18
A-1-13*	0.75	0.5	0.15
A-1-17	2.05		
A-1-20*	0.65	1.15	0.11
A-1-40*	1.39	1.25	1.29
B-1-1*	0.22	1.02	0.22
A-1-41*	8.8	11.3	7.4
A-1-36*	0.71	0.48	0.2
A-1-11*	0.32	0.7	0.13
A-1-21*	1.41	2.7	2.6
A-1-23		0.7	0.06
B-1-8	0.56		
A-1-4	0.06		
A-1-5	1.07		
A-1-9		3.3	2.6
B-2-1		1.27	0.14
Sum	15.87	22.23	12.74
Average	1.32	1.8525	1.96
S.d.	2.39	6.12	2.17

Table S3. Percentage of $CD4^+$ J20/MN cells as a function of time in culture. The percentage of $CD4^+$ cells was measured after 43, 84, and 105 days of culture. High producers are grouped by (a) high or (b) low expression of CD4.

Table S4

A

Days in Culture	27	40	63	78	109	129	150	185	189
A-1-25 (pg/ml)	34,800	84,000	14,400	5,400	270	28	0	0	0
A-1-26 (pg/ml)	109,000	302,000	78,000	100,000	95,000	15,900	6,000	4.4	19.5

B

Days in Culture	39	53	83	109	132	140	189	208	233	260	280
A-1-25 (pg/ml)	>100	>100	>100	98	92	75	25	18			1.1
A-1-26 (pg/ml)	>100	>100	>100	>100	>100	>100	45	40	25	15	0

C

Days in Culture	60	150	210	300
A-1-25 (2-LTR Band)	4988	4930	2688	3536
A-1-26 (2-LTR Band)	6916	6916	2210	1560

Table S4. Relationship between p24 expression and presence of episomal DNA in two moderate producer clones of J20/MN with time in culture. (A-C) The table shows a parallel decline in the expression of p24 in (A, B) two experiments and of (C) 2-LTR circles.

Table S5

Clone	Days in Culture (T+*t*)	Band Area		Ratio
		2-LTR	β-Globin	
A-1-25	60 (T1+*t1*)	4988	6160	0.81
	150 (T1+*t2*)	4930	4928	1.00
	210 (T2+*t2*)	2688	4402	0.61
	300 (T3+*t2*)	3536	5312	0.67
A-1-26	60 (T1+*t1*)	6916	4992	1.39
	150 (T1+*t2*)	6916	4836	1.43
	210 (T3+*t1*)	2210	5852	0.38
	300 (T3+*t2*)	1560	5760	0.27

Table S5. PCR amplification bands of the 2-LTR circles and β-Globin. The intensity of the bands shown in Fig.1 for the PCR amplification of 2-LTR circles and β-Globin of two moderate producers of J20/MN was measured using ImageJ 1.53m software. The ratio of the respective bands is reported.
